# Contextualizing the Norwegian standardized intensity zone framework in an international sample of endurance practitioners

**DOI:** 10.1038/s41598-025-17023-z

**Published:** 2025-10-02

**Authors:** Siren Amelia Seiler-Viken, Fredrik Mentzoni, Stephen Seiler, Sondre Skarli, Thomas Losnegard

**Affiliations:** 1 Department of Physical Performance, Norwegian School of Sport Sciences, Sognsveien 220, 0863 Oslo, Norway; 2 The Norwegian Olympic Sports Center, The Norwegian Olympic and Paralympic Committee and Confederation of Sports, Sognsveien 73, 0854 Oslo, Norway; 3 Faculty of Health and Sport Sciences, University of Agder, Universitetsveien 25, 4630 Kristiansand S, Norway

**Keywords:** Endurance sports, Training intensity, Intensity zones, Heart rate, Zone 2 training, Physiology, Health care

## Abstract

This study investigated potential differences in the adoption and use of endurance intensity zone scales across geographic regions, sports disciplines, practitioner roles, and performance levels. A total of 778 endurance practitioners completed a survey, with 710 responses analyzed for the number of intensity zones used, and 298 responses analyzed for lower percentage of maximum heart rate (%HR_max_) demarcations within a 5-zone scale. A 5-zone scale (47%) was most frequently used. Norwegian respondents were 2.7 times more likely to use a 5-zone scale than all other regions combined. The lower limits of Zones 2–3 differed across regions, and Zones 1–3 across sports, particularly between running and cycling. Norwegian respondents reported the highest mean %HR_max_ lower limits of Zones 2 and 3. Cyclists self-selected the lowest mean %HR_max_ lower limits of Zones 1, 2, and 3. No differences were observed in zone demarcations across roles or performance levels. The results suggest that the standardized intensity scale developed for Norwegian elite endurance athletes has been broadly adopted across sports and performance levels. The widespread use of a single intensity scale is speculated to improve communication across sports. Cyclists may self-select lower zone demarcations in the low-intensity domain by virtue of longer session durations and lower mechanical stress, contributing to the popularity of “Zone 2 training” in cycling.

## Introduction

After deciding modality, the variables that are manipulated to control endurance training load are frequency, duration, and intensity^1^. Training frequency typically refers to the number of training sessions per week (although in some cases it might refer to microcycles of different durations). Duration denotes the length of training sessions or key work periods within sessions in minutes or hours. In the practical prescription of training, intensity can only be interpreted in the context of duration. Intensity zones broadly demarcate differing rates of stress response amplification during training sessions^2^. Monitoring exercise intensity may empower athletes and coaches to manage the magnitude of this stress response from day to day, and optimize adaptive signaling, limit associated systemic stress and enhance performance development over time^3^. While frequency and duration are objectively defined training variables, significant debate arises around the terminology used to describe exercise *intensity* (e.g., “Threshold training”) and the classification of training *zones* (e.g., “Zone 2 training”)^4,5^.

The sports science research literature commonly refers to three physiologically defendable intensity zones demarcated by blood lactate and gas-exchange values^6–16^. Based on these demarcation points, exercise intensity can be divided into low, moderate, or high intensity domains. These three intensity domains differ distinctly in 1) the relative stability of different physiological and perceptual responses when maintaining the intended power or velocity and 2) the duration of continuous exercise tolerated. In trained endurance athletes, the transition between low and moderate intensity typically occurs at a blood lactate concentration of 1.0–2.0 mmol⋅L​^−1^ and is referred to as lactate turn point 1 (LT1)/ventilatory turn point 1 (VT1)^2,5^. The moderate and high intensity domains are demarcated by a second, steeper rise in blood lactate values, typically occurring at around 2.5–4.0 mmol⋅L​^−1^, depending on the athlete’s training status and capacity^17^. This second demarcation is referred to as lactate turn point 2 (LT2)/ventilatory turn point 2 (VT2)^2,5^. The intensity domain between demarcation points LT1 and LT2 may physiologically be described as the “threshold” intensity region^6,18^. The remaining 3^rd^ intensity zone then spans between LT2/VT2 and the lowest intensity eliciting maximal oxygen uptake ($\dot{\text{V}}$O_2max_). In daily coaching practice, three intensity zones may not be nuanced enough as coaches and athletes often desire more narrow intensity training prescriptions. It is therefore common to add additional zone delimitations within the low, moderate, and/or high intensity domains. It can be confusing that these additional zones are arbitrarily/experientially defined and not demarcated with physiologically well-defined “break points”. However, additional zone demarcations, especially within the low- and high-intensity domain, may add precision in the communication around training prescription and response.

Internationally, the 3-zone scale is just one of many used by researchers, coaches, and athletes. Besides the 3-zone scale described above, researchers have employed two intensity zones^19–21^, four intensity zones^22,23^, five intensity zones^3,24–27^, as well as additional “anaerobic” zones above 100% of $\dot{\text{V}}$O_2max_ or the corresponding power/velocity at $\dot{\text{V}}$O_2max_^19,28^. While the literature exhibits reasonable consensus around the physiological demarcation points between Zones 1 and 2, and Zones 2 and 3, no consensus has so far been established on the further subdivision of zones within the 3-zone scale^29,30^.

The prevailing inconsistency in zone methodologies within the scientific literature is amplified in the applied setting where dozens of intensity zone schemes have been proposed^31–34^. On the popular online training platform TrainingPeaks™, coaches and athletes can choose from approximately 40 different intensity scales^35^. These intensity scales define between three and ten zones and can be based on either heart rate percentage ranges, threshold power ranges (functional threshold power; FTP or critical power; CP), pace ranges, or duration ranges. The zone demarcation markers (e.g., percentage of maximum heart rate; %HR_max_) within the scales also vary across scales despite referring to the same relative physiological intensity. Understanding the diversity of intensity scales in the endurance training ecosystem requires some historical context because these scales have been proposed and promoted by individuals across the globe. With the apparent exception of the 3-zone scale, the innovation in the form of guiding intensity scales has not come from sports scientists in the laboratory. It has primarily come from coaches and athletes training and performing daily in the field.

During the 1960 s and 1970 s, the “easy day, hard day” 2-zone system emerged from the influential “Oregon School” of running. Although highly successful and considered revolutionary at the time, this training methodology was not grounded in a formal intensity zone framework or supported by physiological testing. ECG-based heart rate monitors entered the U.S. market as niche products in the mid-1980s. As their popularity grew in the 1990 s, a structured intensity zone nomenclature began to emerge among recreational runners, catalyzed in part by Sally Edwards’ 5-zone scale based on heart rate percentage ranges^31^. In 1998, Jack Daniels introduced a 5-zone scale based on running paces, ostensibly derived from physiological markers, that became highly influential in North American running throughout the 2000s^32^. Around the same time, Joe Friel proposed a 7-zone scale based on lactate threshold heart rate and lactate threshold pace, intended for cycling, mountain bike, and triathlon^36,37^. Andrew Coggan developed the first power-based intensity scale in the early 2000s. His 7-zone scale was based on the power output (Watts) maintained for a set duration (originally 60 min, later as 95% of power held for 20 min). This power output was coined Functional Threshold Power (FTP) and remains popular in cycling as a proxy for power at LT2/VT2 derived from traditional laboratory testing^33^. Meanwhile, in 2000, John Hellemans proposed a 5-zone scale based on blood lactate concentration, heart rate, and subjective perception^34^. After outlining contemporary zone classifications from Europe^38^, Australia^39^, and North America^40^, Hellemans stated that the proliferation of systems made it “…extremely difficult for coaches to understand and use [any] particular classification in practice” [35:6]. Now, 25 years later, the endurance ecosystem appears to have diverged even further from a unified understanding of intensity zones.

**Fig. 1:**
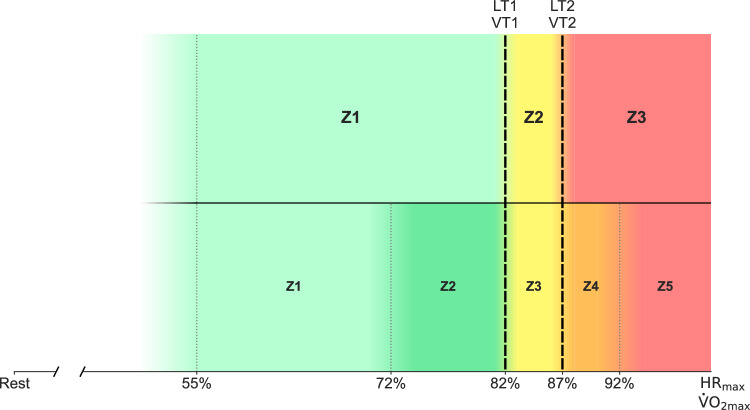
Alignment of the 3- and 5-zone scales anchored by two common lactate/ventilatory turn points (black dashed lines) defining a threshold region (yellow). The figure is adapted from Sitko et al.^5^ with permission. The original version can be found in Seiler^2^. HR_max_ = maximal heart rate; LT1 = lactate turn point 1; LT2 = lactate turn point 2; $\dot{\text{V}}$˙O_2max_ = maximal oxygen uptake; VT1 = ventilatory turn point 1; VT2 = ventilatory turn point 2.

For the last 15–20 years, researchers connected to the Olympic Federation in Norway (Olympiatoppen) have divided the 3-zone scale into five “aerobic” intensity zones with an upper limit of 100% of $\dot{\text{V}}$O_2max_/maximum heart rate (HR_max_) (supplemented with an additional three “anaerobic” zones)^41^. This 5-zone scale is commonly used in data collection and cited by Norwegian scientists in scientific and applied contexts^3,17,24–27^. Using this scale, LT1 and LT2 remain the physiological anchor points but the low-intensity domain (Zone 1) is further divided into Zones 1 and 2, and the high-intensity domain (Zone 3) is split into Zones 4 and 5. Zone 3 in the 5-zone scale aligns with Zone 2 in the 3-zone scale (Fig. 1). The backbone for this scale was first presented in 1995/96 by Rolf Sæterdal and Ørjan Madsen. In collaboration with other scientists and coaches in Norway, they aspired to connect elite athletes across sports through a common understanding of exercise intensity. While the 5-zone scale is not unique to Norway, the Norwegian Olympic Federation appears to be one of the few national governing bodies to have attempted *and* succeeded in implementing a national standardization of intensity zone definitions and markers among elite athletes. However, it is not clear whether this standardized scale is also adopted by sub-elite and recreational athletic populations across different sports, or by extension, how Norwegian athletes and coaches compare with respondents from other geographic regions in the use of training intensity scales.

While numerous intensity methodologies have been proposed and discussed in the literature and applied field, few investigations have explored how these frameworks are used in practice. To promote clearer communication between and among scientists and practitioners, a better understanding of the definitions and applications of different intensity zone scales seems an important step. Therefore, the aim of the present study was to investigate potential differences in the adoption and use of intensity zone scales across geographic regions, sports disciplines, practitioner roles, and performance levels. We hypothesized that Norwegian respondents would be more consistent than athletes and coaches from other geographic regions in the number of zones used, but that the percentage demarcations relative to maximum heart rate within the intensity scale would differ between sports disciplines and performance levels.

## Results

### Number of intensity zones

**Fig. 2:**
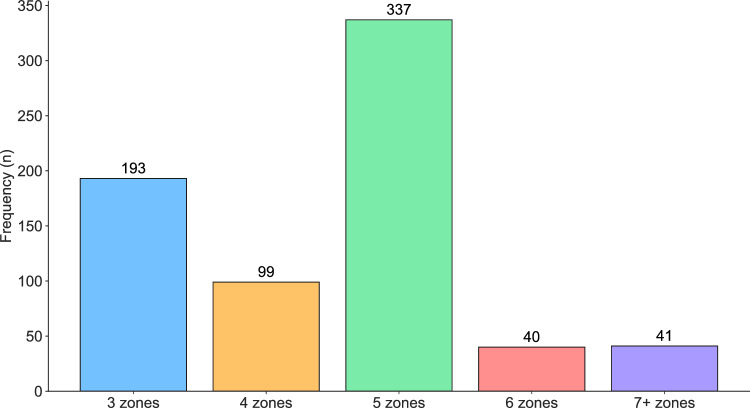
Frequency distribution of the number of intensity zones used in practice by the total sample (*n* = 710).

The distribution of the number of intensity zones utilized by the respondents is illustrated in Fig. 2. The most commonly reported category was ‘5 intensity zones’ ($\frac{337}{710}\approx 47\hspace{0.25em}\text{\%}$), followed by ‘3 intensity zones’ ($\frac{193}{710}\approx 27\hspace{0.25em}\text{\%}$), ‘4 intensity zones’ ($\frac{99}{710}\approx 14\hspace{0.25em}\text{\%}$), ‘7 or more intensity zones’ ($\frac{41}{710}\approx 6\hspace{0.25em}\text{\%}$), and ‘6 intensity zones’ ($\frac{40}{710}\approx 6\hspace{0.25em}\text{\%}$).

### Intensity zones and geographic region

**Fig. 3:**
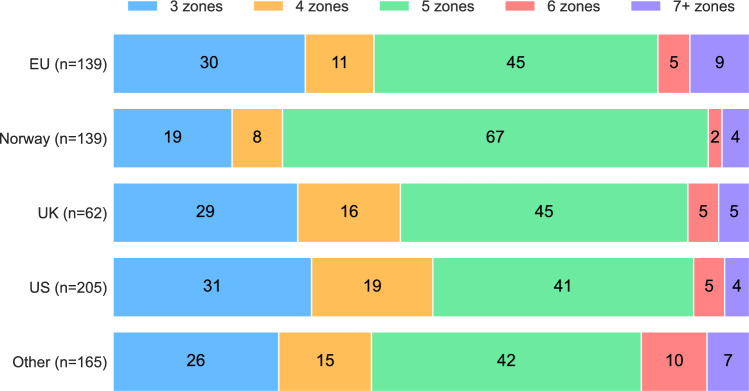
Percentage distribution of the number of intensity zones used across geographic regions (*n* = 710). EU = other European countries; UK = United Kingdom; US = United States.

The relative number (%) of intensity zones used in different geographic regions is illustrated in Fig. 3. Chi-squared tests of independence revealed a significant difference between regions ($p= \times {10}^{-4}, V=0.12$). Pairwise-testing revealed differences in the distribution of the number of intensity zones between Norway and the United States (US) (${p}_{corr}=0.001, V=0.27$), Norway and other countries (Other) (${p}_{corr}=0.003, V=0.27$), and borderline significant difference between Norway and other European countries (EU) (${p}_{corr}=0.05, V=0.23$). All other pairwise comparisons yielded ${p}_{corr}>0.5$.

The odds of using five intensity zones in Norway were $\frac{93}{46}=2.02$, compared to $\frac{244}{377}=0.72$ in the other regions, yielding an odds ratio (OR) of 2.7. This indicates that Norwegian practitioners were 2.7 times more likely to use five intensity zones than practitioners from all other regions combined (Fisher’s exact for 2 × 2 comparison: $p={10}^{-6}, V=0.19$).

### Zone demarcations relative to maximum heart rate

An overview of all mean and standard deviation values for the categories and subgroups analyzed and illustrated in Sects. 2.3.1–2.3.4 are presented below (Table 1).

**Table 1:** Lower demarcation limits across Zones 1–5 for all subgroups, expressed as a percentage of maximum heart rate (%HR_max_) and presented as mean ± standard deviation.

		Zone 1 (%)	Zone 2 (%)	Zone 3 (%)	Zone 4 (%)	Zone 5 (%)
Sport	Cycling	47.3 ± 18.0	65.7 ± 8.2	76.4 ± 6.7	85.9 ± 4.7	92.2 ± 3.2
	Running	55.4 ± 10.2	69.8 ± 6.7	79.6 ± 5.7	86.6 ± 4.1	92.3 ± 3.5
	Skiing	54.2 ± 9.7	69.0 ± 8.5	79.2 ± 6.7	86.3 ± 4.5	92.2 ± 2.9
	Triathlon	51.9 ± 13.8	67.6 ± 5.9	77.5 ± 5.0	85.7 ± 4.5	92.9 ± 3.9
Geography	EU	51.3 ± 15.3	68.6 ± 6.4	78.7 ± 5.4	86.7 ± 3.9	92.5 ± 3.5
	Norway	54.1 ± 10.6	70.2 ± 7.1	80.4 ± 5.4	86.6 ± 3.4	92.1 ± 2.2
	UK	48.6 ± 19.7	66.9 ± 6.0	77.1 ± 5.4	86.0 ± 4.6	92.8 ± 4.6
	US	51.5 ± 14.5	66.7 ± 8.4	77.0 ± 6.9	85.7 ± 5.4	92.5 ± 3.9
	Other	51.7 ± 14.1	66.2 ± 7.6	76.4 ± 6.4	85.7 ± 4.6	92.4 ± 3.7
Level	Tiers 1–2	51.1 ± 15.4	67.9 ± 7.4	77.9 ± 6.2	86.1 ± 4.8	92.3 ± 3.7
	Tiers 3–5	53.2 ± 12.1	68.0 ± 7.6	78.5 ± 6.2	86.3 ± 3.8	92.5 ± 2.9
Role	Athlete	51.0 ± 14.8	67.8 ± 7.5	78.1 ± 6.1	86.1 ± 4.4	92.3 ± 3.5
	Coach	55.0 ± 11.8	68.9 ± 6.3	78.8 ± 5.2	86.6 ± 3.8	92.7 ± 3.2

ata are based on a subset of the original 710 survey respondents who reported using a 5-zone scale and categorized themselves as cyclists, runners, cross-country skiers/biathletes, or triathletes (*n* = 298; Fig. 4,5,6,7).

Values are presented as mean ± standard deviation.

**Fig. 4:**
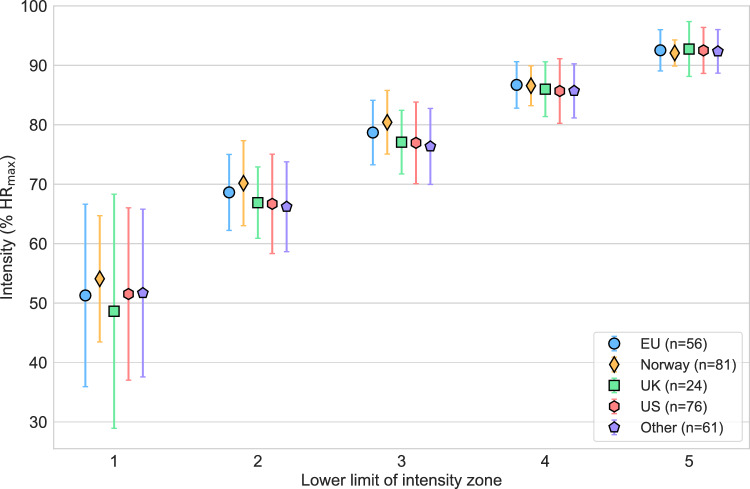
Estimation of the lower limit of intensity Zones 1–5 (relative to maximum heart rate) organized by geographic region. Mean (markers) and standard deviation (error bars) are indicated. EU = other European countries; UK = United Kingdom; US = United States; %HR_max_ = percentage of maximum heart rate.

**Fig. 5:**
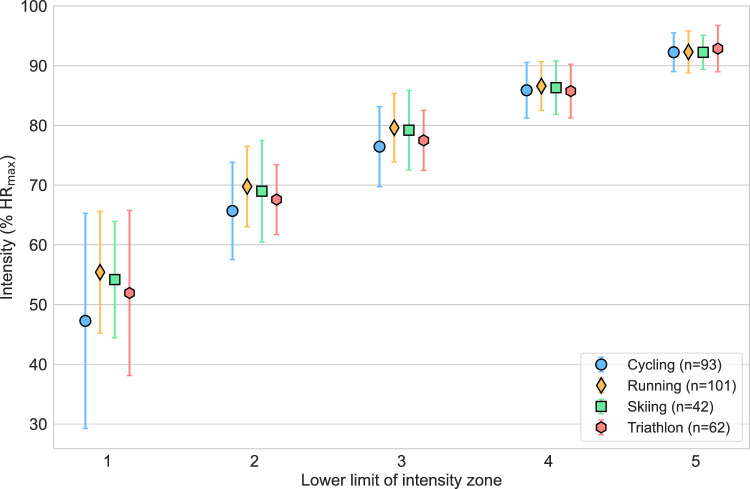
Estimation of the lower limit of intensity Zones 1–5 (relative to maximum heart rate) organized by sport. Mean (markers) and standard deviation (error bars) are indicated. Cycling = road- and mountain cycling; Running = middle-, long-, and ultra-distance running; Skiing = cross-country skiing and biathlon; %HR_max_ = percentage of maximum heart rate.

**Fig. 6:**
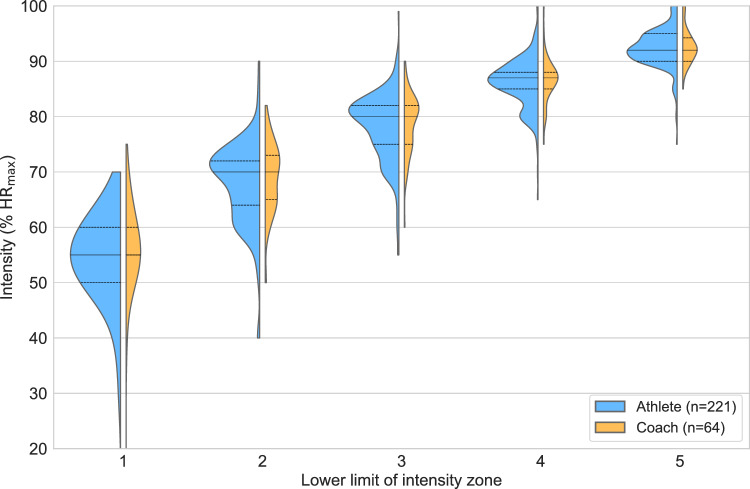
Kernel density estimation of the lower limit of intensity Zones 1–5 (relative to maximum heart rate) split by role; athlete vs. coach. Median (solid line) and upper and lower quartiles (dashed lines) are indicated. %HR_max_ = percentage of maximum heart rate.

**Fig. 7:**
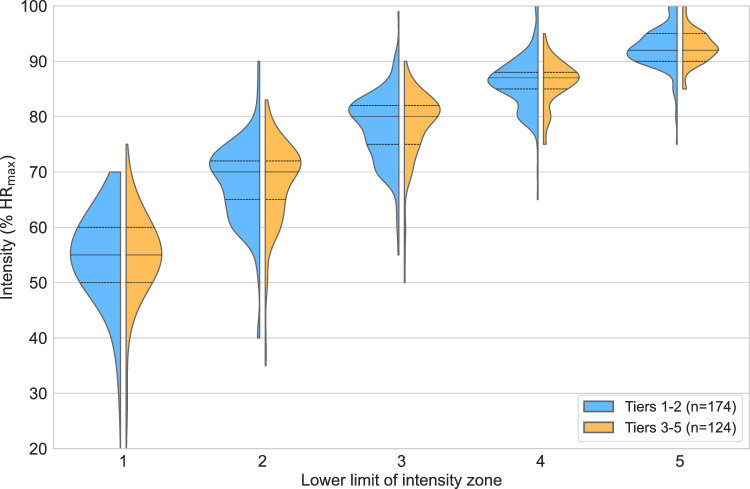
Kernel density estimation of the lower limit of intensity zones 1–5 (relative to maximum heart rate) split by level; low (Tiers 1–2) vs. high (Tiers 3–5). Median (solid line) and upper and lower quartiles (dashed lines) are indicated. %HR_max_ = percentage of maximum heart rate.

#### Lower intensity zone limit dependence on geography

The dataset was categorized into five geographic regions. There were 56 respondents from EU (France [11], Sweden [11], Italy [9], Germany [8], Netherlands [6], Spain [5], Denmark [4], Finland [2]), 81 from Norway, 24 from the United Kingdom (UK), 76 from the US, and 61 from ‘Other’ (Australia [10], Canada [12], Other [38]). Shapiro–Wilk tests showed normality in only 3 of 25 zone-sport combinations (UK: Zones 2–3 and Other: Zone 4). Kruskal–Wallis tests revealed significant differences between geographic regions in the lower limit of Zones 2 ($p={10}^{-5}$) and 3 ($p={10}^{-8}$), but not in Zones 1 ($p=0.6$), 4 ($p=0.4$) or 5 ($p=0.7$).

The observed differences were between Norway and Other (Zone 2: $p$-value, ${p}_{corr}={10}^{-4}$ $g=0.54$; Zone 3: ${p}_{corr}={10}^{-7}$, $g=0.69$), between Norway and the UK (Zone 2: ${p}_{corr}=0.03$, $g=0.47$; Zone 3: ${p}_{corr}=0.004$, $g=0.62$), between Norway and the US (Zone 2: ${p}_{corr}=0.001$, $g=0.45$; Zone 3: ${p}_{corr}= \times {10}^{-6}$, $g=0.56$), and between Norway and EU (Zone 3: ${p}_{corr}=0.02$, $g=0.32$). All other pairwise comparisons yielded ${p}_{corr}>0.4$. An illustration of the difference between geographic regions is presented in Fig. 4.

#### Lower intensity zone limit dependence on sport

The dataset included 93 cyclists, 101 runners, 42 skiers (cross-country and biathlon), and 62 triathletes. Shapiro–Wilk tests revealed that none of the 20 combinations of zone and sport were likely normally distributed (all $p<0.002$). Kruskal–Wallis tests revealed significant differences between sports in the lower limit of Zones 1 ($p=0.001$), 2 ($p={10}^{-5}$) and 3 ($p={10}^{-6}$), but not in Zones 4 ($p=0.1$) or 5 ($p=0.8$).

The observed differences were between running and cycling (Zone 1: ${p}_{corr}={10}^{-4}$, $g=-0.56$; Zone 2: ${p}_{corr}={10}^{-4}$, $g=-0.54$; Zone 3: ${p}_{corr}={10}^{-5}$, $g=-0.51$), between cycling and skiing (Zone 2: ${p}_{corr}=0.002$, $g=-0.40$; Zone 3: $0.001$, $g=-0.41$), between running and triathlon (Zone 2: ${p}_{corr}=0.04$, $g=0.34$; Zone 3: ${p}_{corr}=0.005$, $g=0.38$), and between skiing and triathlon (Zone 3: ${p}_{corr}=0.01$, $g=0.30$). All other pairwise comparisons yielded ${p}_{corr}>0.05$. An illustration of the difference between sports groups is presented in Fig. 5.

#### Lower intensity zone limit dependence on role

The dataset consisted of 221 athletes, 64 coaches, and 13 sports scientists. Shapiro–Wilk tests showed normality in only 3 of 15 zone-role combinations, all within the relatively small sports scientist group (Zones 3, 4, and 5). Kruskal–Wallis tests revealed no significant differences between roles in any zone (all $p>0.1$). An illustration of the difference between athletes and coaches is presented in Fig. 6.

#### Lower intensity zone limit dependence on level

There were 39 respondents in Tier 1, 135 in Tier 2, 77 in Tier 3, 34 in Tier 4, and 13 in Tier 5. In addition to comparing the five tiers to each other, we differentiated between the two lowest levels (Tiers 1–2, $n=174$) and the three highest levels (Tiers 3–5, $n=124$) in the present analysis. An illustration of the difference between the lowest level (Tiers 1–2) and the highest level (Tiers 3–5) is presented in Fig. 7.

Shapiro–Wilk tests indicated that none of the 25 combinations of zone and tier, or the 10 combinations of zone and lowest vs. highest level, were likely normally distributed (all $p<0.01$). Kruskal–Wallis tests revealed no significant differences between the Tiers 1–5 in any zone (all $p>0.1$), or for the lowest vs. highest level (all $p>0.2$).

## Discussion

Training intensity zones and training intensity distribution currently attract substantial research attention. In the present study, we attempted to 1) quantify intensity scale use and 2) explore potential sport specific or other explanatory aspects of the use patterns seen. We report three key findings. First, the number of intensity zones ranged from 3 to 7 + among respondents, with a 5-intensity zone scale as the most used. Second, our results revealed that Norwegian respondents showed a 2.7 × greater likelihood of using a 5-zone scale compared with all other geographic regions, consistent with the national intensity zone framework that has been developed in Norway. Finally, a sport specific discrepancy in the application of the 5-zone scale was observed. Cyclists self-selected significantly lower demarcation points for the lowest three intensity zones in the 5-zone scale than all other sports. We observed no systematic differences in zone demarcations provided by coaches versus athletes, or across the five performance tiers from McKay et al.^42^.

To our knowledge, this is the first study to quantify the use of different intensity scales in the training and monitoring of endurance athletes, coaches, and scientists. Numerous intensity scales have been developed in the applied field^31–41^, but the rigor of validation is not clear. An intensity scale represents a communication tool and calibration framework in a continuous training monitoring process. Intensity zone systems often evolve as a combination of physiology and pedagogy. Physiologically, there is reasonable consensus around the testing-based identification of two ventilatory and blood lactate turn points that provide demarcations for three intensity zones^6–9^. The 5-zone scale reported as preferred in this group of respondents aligns with the physiologically anchored 3-zone scale via a common threshold region. The two additional zones in the 5-zone scale are created by arbitrarily sub-dividing the intensity region below LT1/VT1 and the intensity region above LT2/VT2 but below $\dot{\text{V}}$˙O_2max._ The resulting four narrower intensity zones below and above the shared threshold intensity zone (Fig. 1) are utilized to support more precise prescription and monitoring.

In the endurance ecosystem, we argue that intensity scale development has mostly been ad hoc. A known exception to this norm is the attempted standardization of a national 5-zone scale in Norway. Over the past two and a half decades, a common intensity scale has been developed and used to facilitate comparison of training characteristics and promote meaningful discussions among athletes, coaches, and scientists that train and work under the umbrella of the Norwegian Olympic Federation (Olympiatoppen). However, no former investigations have attempted to quantify whether this framework scales down to the sub-elite and recreational athletic populations across different sports. The results of the present survey suggest that this approach has indeed facilitated greater cohesion around a common intensity scale in Norway across performance tiers and sports compared to other regions (Fig. 3), with 67% of Norwegian respondents utilizing five zones and 19% utilizing three zones.

The Norwegian 5-zone scale was developed based on an integration of years of research and laboratory testing of thousands of athletes, field testing, anecdotal evidence, and critical discussions. The original values for blood lactate, heart rate, and oxygen consumption demarcating each zone were based on physiological test results from Sæterdal and Madsen’s own scientific research and practical experience with elite athletes. Their proposals were also greatly influenced by prominent German exercise physiologists of the time, in particular the work of Alois Mader and colleagues^30,43–46^. Ever since its practical implementation in the early 2000 s, the 5-zone intensity scale has been an essential tool in the development of Norwegian elite athletes and is today internationally recognized for its defensible scientific foundation and practical utility^2,3,17,24–27,41^. We speculate that this common communication platform (“intensity zone language”) has played a positive role in the relative success of Norway across a broad range of endurance sports^25^. A shared intensity framework with physiological demarcation points enables comparisons across sports and enhances communication between and among coaches, athletes, and scientists.

Given that the intensity scales typically include arbitrary demarcations, it is natural to assume that these demarcations expressed as %HR_max_ or other parameters are also arbitrary. A second part of this study was to investigate potential differences in the self-selected lower demarcations of intensity zones 1–5 based on %HR_max_. We found that the greatest degree of variation in responses was in the low-intensity domain (up to the start of Zone 3). As intensity increased, the variability in percentage demarcations among respondents decreased, aligning with the lower variation in typical work duration within higher-intensity zones. This was apparent for the differences between geographical regions (Fig. 4) and sports (Fig. 5).

The greater variability in zone demarcations in the low-intensity domain can be attributed to different factors. First, differences in the relative contribution of resting heart rate to a given percentage of HR_max_ is a source of inter-individual variability. An athlete with a resting heart rate of *40* beats per minute (BPM) and a maximum heart rate of 190 BPM has a larger heart rate reserve (HRR) compared to a person with a resting heart rate of *55* BPM and a maximum heart rate of 190 BPM. Accordingly, percentage of HRR correlates more strongly with percentage of $\dot{\text{V}}$˙O_2max_ than %HR_max_^47,48^. The variability associated with this source of individual variation decreases as the exercise intensity increases. The same is true for blood lactate concentration measurements as a function of work intensity. The same absolute difference of 0.5 mmol·L^−1^ between 1.2 and 1.7 mmol·L^−1^ (~ 42%) is much more problematic when interpreting lactate profiles than the difference between 7.8 and 8.3 mmol·L^−1^ (~ 6%). The same is also true for RPE. Athletes show greater variability in ratings of perceived exertion at low intensities compared to higher intensities^17^. As we progress towards the high intensity zones, the relative error of measurement of these markers decreases^49^.

Another factor contributing to the discrepancy of zone demarcations within the 5-zone scale was the lower zone demarcations among cyclists in the low- to moderate-intensity domain compared to all other sports (Fig. 5). In contrast to running, cycling is non-weight bearing with frequent brief recovery bouts during typical training and racing situations. This is due to both periods of drafting behind other riders in a group and coasting downhill after climbing. For example, a sample of power data (1 Hz sampling frequency) from 50 one-day races and stages of Tour and Grand Tour races distributed among six riders from four World Tour teams showed that 15–30% of total race duration was spent at a power output below 25 Watts [S. Seiler, unpublished data, March 2025]. Power output is highly stochastic in cycling by virtue of these differences. While technically demanding, these natural “rest periods” tend to reduce mean heart rate responses during continuous cycling. Heart rate variation is smaller during running on the same terrain, where the downhill sections involve eccentric motion that places significant demands on muscular effort and metabolic energy production, thereby maintaining a continuous higher internal intensity. Because of these intrinsic differences in movement patterns and muscle actions, the typical duration of long, low intensity sessions differs vastly between the two modalities^25^.

In this context, intensity cannot be viewed in a vacuum. It is the product of intensity *and* duration that induces both the adaptive signal and the acute, dynamically evolving systemic stress responses during any training session^2^. A professional road cyclist’s Zone 1 training session will often extend beyond 6 h, while a 2-h Zone 1 training session is considered prolonged for an elite runner. The lower self-selected limit for the start of Zone 1 by cyclists (Fig. 5) may result from longer session durations and more integrated periods of near-zero power output. Similar stochasticity is seen in cross-country skiing due to periods of gliding on downhills and behind other athletes on flat sections^50^. The current discussion and debate around Zone 1 and Zone 2 largely emerged from the sport of road cycling, with very low mechanical load and low-intensity, long duration training sessions^5^. Consequently, because we lack common terminology for the different intensity zones and zone demarcations, Zone 2 heart rate for a cyclist may correspond to Zone 1 heart rate for a runner, even though the relative intensity is the same. Consistent with our findings, Tjelta and Enoksen^51^ accounted for modality-specific differences by setting the lower limit of Zone 1 in a 5-zone scale at 55% of HR_max_ for cycling and 60% of HR_max_ for running. In contrast, respondents in the present study selected lower mean Zone 1 limits: approximately 47% and 55% of HR_max_, for cyclists and runners, respectively. A testable hypothesis that emerges from this study is that cycling at 55% of HR_max_ for four hours induces the same adaptive signal *on average* as cycling at 70% of HR_max_ for two hours.

While the agreement in zone demarcations between athletes and coaches (Fig. 6) was expected due to the organic communication and training philosophy alignment that emerges, we were surprised by the lack of difference in zone demarcations across performance levels (Fig. 7). While the 3-zone scale is rooted in reproducible, albeit fuzzy, physiological markers, additional zone demarcations within the low-intensity domain (Zones 1–2) and high-intensity domain (Zones 4–5) are arbitrary and not definitionally anchored in specific physiological or psychometric transitions or events^27^. The corresponding heart rate percentages of ~ 80% and ~ 88% of the maximal heart rate achieved during a training session (HR_peak_) at LT1 and LT2, respectively, are based on data from highly trained and elite athletes^17^. These guidelines are therefore likely to strongly overestimate the “threshold range” for beginner and recreational athletes^26,52,53^. Because of this, the scaling of exercise intensity based on %HR_max_ will differ between individuals of different training and performance levels. While a common “intensity zone language” is warranted at the group level, one must recognize that the relative percentage of maximum heart rate at any training zone will depend on both short- and long-term training characteristics and should ideally be established with individual testing. An elite athlete’s heart rate at LT2 may correspond to 90% of HR_max_^17^, while a beginning runner’s or cyclist’s heart rate at LT2 may initially correspond to 75–80% of HR_max_. This discrepancy, combined with large variation in resting and maximal heart rate independent of age, makes heart rate an intensity tool that can easily be misinterpreted and misused without individualized testing.

This study is the first to investigate the practical use of intensity zone scales in endurance sports. However, the lack of prior research limits comparison. Several other limitations should also be acknowledged. First, this was a cross-sectional study based on survey responses. The survey was shared by the authors via X (formerly Twitter), targeted e-mail correspondence, Facebook, and Instagram. Selection bias was likely present regarding sex (80% male respondent), geographic origin, and sports affiliation. Consequently, our sample was not globally representative due to the social media recruitment strategy and the English language limitation. The sample has an overrepresentation of Norwegian respondents compared to other subgroups and may have an underlying bias towards the 5-zone scale. Further, ‘Other’ was included as an answer option to questions 4 (role) and 5 (sport). These responses were not included in the final analysis as they could not be grouped into any category. We also acknowledge that the survey questions were not validated but based on standardized intensity zone frameworks used in peer-reviewed literature (i.e., the 3- and 5-zone scale), as well as the intensity scales available online employed by popular training platforms and software developers. Finally, the authors had unique insight into the origins and use of the standardized intensity scale used in Norway. We therefore chose to explore intensity zone use among Norwegian endurance practitioners as a special case that was hypothesized to contrast to the more ad hoc development of intensity zones in other countries. Because of this focused attention on the Norwegian approach to training intensity zones, the article cites several studies performed by Norway based sport scientists with Norwegian elite endurance athletes as common data sources.

## Conclusion

The scientific literature commonly refers to three physiological intensity zones. Our findings suggest that endurance practitioners seek greater precision around training prescription and response, as a 5-zone intensity scale was most widely used. Because these additional intensity zone demarcations are arbitrary, selected boundary values for variables such as %HR_max_ are also largely self-selected. We found that cyclists, who are characterized by employing longer low-intensity endurance sessions compared to athletes in other endurance sports, also self-selected lower minimum limits for intensity Zones 1–3 in a 5-zone scale. This is consistent with greater intensity x duration variation in cycling and may help explain the origins of the heated debate around the importance of “Zone 2 training”. Finally, this study provides evidence that the development of a standardized intensity scale in Norway has resulted in marked adoption of this scale across sports and performance levels. The Norwegian Olympic Federation and cooperating sport scientists have standardized the intensity scale with the expressed intent to improve communication across sports and enhance organizational knowledge sharing. Whether this strategy has contributed to international performance success is a matter of speculation.

## Methods

### Participants and study design

This was a cross-sectional study based on self-reported survey data. The target population were individuals actively engaged in endurance sports with prior experience in training intensity monitoring. 778 individuals across all age groups (15–70 + years) responded to the English language survey which was shared via social media platforms (X, Facebook, Instagram), online forums, and targeted email invitations. The sample was composed of athletes, coaches, and sports scientists of both sexes practicing one of the following endurance sports: Road/track/mountain cycling, middle-distance/distance/ultra-distance running, swimming, triathlon, cross country skiing or biathlon, short/long track speed skating, rowing, kayak/canoe, or ‘Other’ than the ones listed. The respondents reported the following home countries: Australia, Canada, Denmark, Finland, France, Germany, Italy, Netherlands, New Zealand, Norway, Spain, Sweden, United Kingdom, USA, or ‘Other’ than the ones listed. The respondents self-reported their performance level according to five predefined tiers (Tier 1: Recreationally Active, Tier 2: Trained/Developmental, Tier 3: Highly Trained/National Level, Tier 4: Elite/International Level, Tier 5: World class) from McKay et al.^42^. Participants were encouraged to respond to the survey if they: 1) were actively engaged in endurance sports, 2) monitored exercise intensity during training and/or competition, and 3) used intensity scales to define intensity zones.

In the present study, we focused on the use of “aerobic” intensity zones (up to 100% of HR_max_) in practice, with further focus on practical intensity demarcations (relative to maximum heart rate) within the 5-zone scale. The following filtering steps were performed before the analysis of the number of intensity zones used in practice (Fig. 2–3).

- Removed 11 responses that did not practice an endurance sport (*n* = 767).
- Removed 57 responses that could not be categorized as athletes, coaches, or sports scientists (*n* = 710).

Further refinement of the data was conducted before the analysis of lower %HR_max_ demarcations within the 5-zone scale:

- Removed 373 responses that did not use five intensity zones, cf. Figure 2 (*n* = 337).
- Removed 7 responses with erroneous heart rate zone input (*n* = 330).
- Removed 32 responses that could not be categorized as cyclists, runners, cross-country skiers/biathletes, or triathletes (*n* = 298).

Our dataset therefore has a two-part structure: 1) all survey respondents who practiced an endurance sport and could be categorized as athletes, coaches, or sports scientists (n = 710; Fig. 2, 3), and 2) a subset of these 710 survey respondents who utilized the 5-zone scale and categorized themselves as cyclists, runners, cross-country skiers/biathletes, or triathletes (n = 298; Fig. 4,5,6,7).

### Survey structure and questions

A structured survey consisting of 29 questions was developed in Google Forms (Google LLC, California, USA) to collect quantitative data on the use of intensity scales across countries, endurance sports, practitioner roles, and performance levels. The questions were developed based on existing literature on exercise intensity zones (i.e., the 3-zone scale demarcated by the first and second lactate/ventilatory threshold; LT1/VT1 and LT2/VT2)^6–9^, exercise intensity scales from online platforms (TrainingPeaks™^35^, Intervals.icu) and software developers (Polar™^54^, Garmin™^55^), and the authors’ expertise in applied endurance sports.

The survey was divided into 4 sections: 1) Descriptive characteristics (questions 1–7), 2) Exercise intensity quantification (questions 8–15), 3) Standardized intensity scales/frameworks (questions 16–23), and 4) The 5-zone intensity scale (questions 24–29). The first 7 questions assessed descriptive characteristics of the responders, including age, sex, sport, country, role, and level (Tiers 1–5 based on criteria from McKay et al.^42^. Questions 8–11 were focused on the organization of intensity scales (the number of zones used in the “aerobic” and “anaerobic” range, how the intensity zones were determined, the source(s) for the intensity scale used). Questions 12–15 assessed the parameters used to monitor training intensity (heart rate, power, velocity/pace via GPS/GNSS or other method, blood lactate concentration, ventilation, rating of perceived exertion; RPE, subjective “feeling”, or ‘Other’) and the metric(s) underlying the intensity scale used (% of maximal heart rate; HR_max_, % of HR_max_ at lactate threshold; %HR LT, functional threshold power; FTP, critical power; CP, threshold speed and additional speed zones, pace relative to time/distance, ventilation markers; VT1/VT2, blood lactate markers; LT1/LT2, or ‘Other’). Question 16–23 assessed the use of standardized intensity scales/frameworks available on popular online training platforms and from software developers. Questions 24–28 aimed to quantify the %HR_max_ demarcations of predefined intensity domains characterized as Zone 1 (easy/recovery), Zone 2 (easy/steady), Zone 3 (moderate/threshold), Zone 4 (hard/severe), Zone 5 (very hard/extreme). The respondents were encouraged to comment the starting point in percentage of HR_max_ for each respective intensity zone (1–5) (Fig. 1). Question 29 aimed to evaluate the practical application of intensity quantification variables within the Norwegian Olympic Federation’s intensity scale^41^.

A pilot test was conducted with 4 individuals to confirm the verbal clarity of the questions, acceptable time usage, and the functionality of the Google Forms format.

### Survey variables

The survey included both categorical and numerical variables. The main variables of interest in the present analysis include: 1) The number of intensity zones used within the “aerobic” range (up to 100% of HR_max_), and 2) the lower %HR_max_ limits of intensity zones 1–5 within the 5-zone scale. Independent variables of interest with respect to the main outcome were: 1) Geographic region, 2) Sports discipline, 3) Practitioner role, and 4) Performance level. The geographic regions were categorized as Norway, the United States (US), the United Kingdom (UK), other European countries (EU), and other countries (Other). The sports were categorized as road- and mountain cycling (Cycling), middle-, long- and ultra-distance running (Running), cross-country skiing and biathlon (Skiing), and triathlon (Triathlon). The roles were categorized as ‘Athlete’, ‘Coach’, and ‘Sports scientist’. The performance levels were categorized as recreationally active (Tier 1), trained/developmental (Tier 2), highly trained/national level (Tier 3), elite/international level (Tier 4), and world class (Tier 5). Most questions required categorical responses, where participants selected from predefined categories. The lower limits of intensity zones 1–5 within the 5-zone scale were measured numerically. All questions, except for an open-ended question that was not relevant to the present study, were marked as mandatory (*), meaning that survey progression was halted until an answer was provided. The survey is available as supplementary material in an external repository^56^.

### Data security and subject anonymity

The study was carried out according to the Declaration of Helsinki and data collection methods were performed according to the guidelines of the Norwegian Centre for Research Data. As the study involved fully anonymous, non-interventional survey data and did not constitute a clinical trial, formal pre-registration was not required under the Declaration of Helsinki. The study was reviewed by the Ethics Committee of the Norwegian School of Sport Sciences and found to not require full ethical review due to the completely anonymous nature of the data collection and absence of health-related or other personally identifiable information. In accordance with the regulations of the Norwegian School of Sport Sciences, this project received expedited ethical approval from the Chair of the Ethics Committee. Participants in the study were informed that the survey was conducted for research purposes, and that all data collected would remain fully anonymous, with no digital links to or records of the personal identity of participating subjects. Completion and submission of the digital survey was therefore taken as informed consent. According to the Health Research Act in Norway, adolescents above the age of 15 years may consent to participate in research projects that do not involve the collection of sensitive or personal information. Completion and submission of the digital survey was therefore taken as informed consent. The survey remained open for data collection from 13.03.2024 to 07.08.2024 (145 days).

### Analysis

Python 3.11 was used for data processing and analysis. The *pandas* and *pingouin* packages were employed for numerical and statistical analyses, whereas *matplotlib*, *seaborn*, and *squarify* were used for generating figures. The statistical analyses were also verified using R 4.4.2.

Chi-squared tests of independence were conducted to examine differences in the number of intensity zones used across geographic regions (Fig. 3). Shapiro–Wilk tests were performed to assess normality of each lower limit of zones 1–5 filtered by geographic regions (Fig. 4), sports disciplines (Fig. 5), practitioner roles (Fig. 6), and performance levels (Fig. 7). Kruskal–Wallis tests were performed to assess differences in the lower limit of intensity zones 1–5 between geographic regions (Fig. 4), sports disciplines (Fig. 5), practitioner roles (Fig. 6), and performance levels (Fig. 7). To account for multiple comparisons in the pairwise tests, we applied a Bonferroni correction to control the family-wise error rate for both Chi-squared and Kruskal–Wallis tests.

Effect sizes were estimated using Cramér’s V and Hedges’ g. Cramér’s V is proportional to chi and reflects the strength of the relationship on a scale from 0 (no association between the variables) to 1 (perfect association). Hedges’ g is a modification of Cohen’s d that includes a correction factor, making it more appropriate for small or unequal sample sizes.

## Data Availability

The datasets generated and analyzed during the present study are available in the “Contextualizing the Norwegian standardized intensity zone framework in an international sample ofeEndurance practitioners” repository on Figshare 10.6084/m9.figshare.c.7711532^56^.
